# *In vitro* activity of imipenem/relebactam and comparators against *Enterobacterales* isolates collected in Brazilian hospitals according to results from the Study for Monitoring Antimicrobial Resistance Trends, 2020–2021

**DOI:** 10.1128/spectrum.02543-24

**Published:** 2025-06-05

**Authors:** Amanda Azevedo Bittencourt, Vinicius Lima Faustino, Paula de Mendonça Batista, Lays Paulino Leonel, Marina Della Negra de Paula, Thales José Polis

**Affiliations:** 1 Global Medical & Scientific Affairs (GMSA), MSD Brazil 67939 , São Paulo, Brazil; 2 Real World Evidence, IQVIA Brazil, São Paulo, Brazil; JMI Laboratories, North Liberty, lowa, USA

**Keywords:** antibiotics, bacterial infections, *Enterobacterales*, imipenem/relebactam, surveillance

## Abstract

The Study for Monitoring Antimicrobial Resistance Trends (SMART) surveillance program tested imipenem/relebactam (IMR) and comparators against a total of 2,258 non-duplicate clinical isolates of *Enterobacterales (Klebsiella pneumoniae* and *Escherichia coli*) collected across six Brazilian cities during 2020–2021. Antimicrobial susceptibility was determined by the Clinical and Laboratory Standards Institute (CLSI) reference broth microdilution method and interpreted following Brazilian Committee on Antimicrobial Susceptibility (BrCAST)/European Committee on Antimicrobial Susceptibility Testing (EUCAST) breakpoints. *Enterobacterales* isolates were screened for β-lactamase genes (*bla*) by sequencing. The most frequent isolates identified among *Enterobacterales* were *E. coli* (*n* = 471; 20.9%) and *K. pneumoniae* (*n* = 453; 20.1%). Susceptibility testing showed that >99% of *E. coli* isolates were susceptible to colistin ([COL], *n* = 470; 99.8%), meropenem ([MEM], *n* = 468; 99.4%), IMR (*n* = 468; 99.4%), and ceftazidime/avibactam ([CZA], *n* = 468; 99.4%), whereas a high proportion of *E. coli* multidrug-resistant (MDR) isolates were susceptible to COL (*n* = 69; 98.6%), CZA (*n* = 67; 95.7%), MEM (*n* = 67; 95.7%), IMR (*n* = 67; 95.7%), and amikacin ([AMK], *n* = 66; 94.3%). Overall, *K. pneumoniae* isolates were most susceptible to CZA (*n* = 414; 91.4%) and IMR (*n* = 411; 90.7%) and over 86.0% of *K. pneumoniae* MDR isolates were susceptible to CZA (*n* = 268; 87.3%) and IMR (*n* = 265; 86.3%). This study reported that IMR was one of the most effective *in vitro* β-lactam–β-lactamase inhibitor combinations tested and demonstrated comparable activity to CZA and higher activity than COL against both the *Enterobacterales* species tested.

**IMPORTANCE:**

As new mechanisms of antibiotic resistance continue to emerge, especially with the rise of carbapenem resistance, this study emphasizes the importance of assessing the current landscape of antimicrobial resistance to identify optimal therapeutic approaches. In this way, the publication of national data is significant for understanding local epidemiology to evaluate new drugs and make the best choice among the antimicrobials available in the hospital environment, both for critically ill patients with resistant bacterial infections.

## INTRODUCTION

Multidrug resistance (MDR) among Gram-negative bacterial infections has become a serious public health concern worldwide (1, 2), with carbapenem-resistant *Enterobacterales* (CRE) being of particular concern. Carbapenems are highly potent, and the most commonly used β-lactam antibiotics for the treatment of infections caused by MDR *Enterobacterales*, including *Escherichia coli* and *Klebsiella pneumoniae* (3). However, the emergence and spread of CRE are an important cause of hospital-associated infections, leading to high clinical failure and mortality (4, 5). CRE shows geographical variations in prevalence and predominates in many countries, including Brazil (6–9). According to the Brazilian Health Regulatory Agency, in Portuguese, *Agência Nacional de Vigilância Sanitária* (ANVISA), >40.0% of *K. pneumoniae* and *Pseudomonas aeruginosa* isolates causing catheter-related blood infections in intensive care unit adult patients are resistant to carbapenems (10).

There are three major mechanisms responsible for CRE: enzyme production, overexpression of efflux pumps, and decreased cell-membrane permeability (1). Of these, enzyme production is the main resistance mechanism. Enzymes are categorized into three groups: KPC (*K. pneumoniae* carbapenemase) (Ambler class A), MBLs (metallo-ß-lactamases) (Ambler class B), and OXA-48-like (Ambler class D). One of the most common mechanisms of CRE is the production of KPC, which hydrolyzes and inactivates β-lactam antibacterial agents (3–5). Although KPC was initially found in *K. pneumoniae* isolates, isolates of KPC-producing *E. coli, Klebsiella oxytoca, Salmonella enterica, Citrobacter freundii, Enterobacter aerogenes, Enterobacter cloacae, Proteus mirabilis,* and *Serratia marcescens* have also been identified (11). *Enterobacterales* that produce KPCs are highly prevalent and have acquired resistance to β-lactams, which limits antimicrobial options for treatment.

Imipenem (IPM) is a broad-spectrum carbapenem (β-lactam) antibiotic that inhibits cross-linking of peptidoglycans during bacterial cell-wall synthesis by inactivating penicillin-binding protein, leading to cell lysis and death (12). Relebactam is an inhibitor of class A and class C β-lactamases that protects IPM from degradation by Amber class A and class C β-lactamases including *Pseudomonas*-derived cephalosporinase (12). Imipenem/relebactam (IMR) is a recently approved (Food and Drug Administration [FDA] 2019; European Medicines Agency 2020; ANVISA 2024) novel β-lactam–β-lactamase inhibitor combination that has shown great potential in the treatment of complicated urinary tract infections, complicated intra-abdominal infections, hospital-acquired pneumonia, and ventilator-associated pneumonia caused by many CRE strains (13, 14). IMR has expanded microbiologic activity against MDR Gram-negative pathogens, including carbapenem-resistant non*-Morganellaceae Enterobacterales* (NME) and difficult-to-treat resistant *P*. *aeruginosa* (15). The FDA and Clinical and Laboratory Standards Institute (CLSI) IMR susceptibility breakpoint for *Enterobacterales* is ≤1/4 mg/L, while the European Committee on Antimicrobial Susceptibility Testing (EUCAST) breakpoint is ≤2/4 mg/L (16, 17). Antimicrobial resistance surveillance programs serve many different functions such as identification and detection of novel resistance phenotypes and delineating mechanisms of resistance; evaluating and predicting the trends in resistance; monitoring the effectiveness of new antimicrobials in clinical use; tracking and reporting outbreaks of resistant organisms; assisting infection control and guiding public health programs; and generating data for new drug applications and/or other submissions to regulatory agencies (18).

Previous studies conducted worldwide consistently have reported the good *in vitro* activity of carbapenems against clinical isolates of *Enterobacterales* over time (10, 19–22). However, there has been a concerning increase in the prevalence of carbapenemase and β-lactamase enzyme production, coupled with alterations in permeability, leading to the development of carbapenem resistance (23). This situation highlights the critical need for ongoing surveillance of antimicrobial susceptibility, especially for carbapenems.

Global surveillance programs such as Study for Monitoring Antimicrobial Resistance Trends (SMART) play a crucial role in evaluating the antimicrobial susceptibility of Gram-negative bacilli species, collected from a wide range of samples. The SMART study is a worldwide program designed to monitor longitudinally the involvement of aerobic and Gram-negative bacteria, both from community and nosocomial acquisition, as well as their patterns of resistance. The program is associated with multiple published articles representing the general picture of antimicrobial susceptibility in many countries (10, 21, 22, 24). The latest study from the US provided updates up to 2020 on MDR-producing isolates from the lower respiratory and urinary tracts, intra-abdominal region, and blood (21). The SMART program also processes many bacteria providing the opportunity to identify and track novel resistance mechanisms.

This study provides an update on antimicrobial susceptibility testing results for *Enterobacterales* isolates submitted to the SMART surveillance program from 2020 to 2021 by 10 study sites in Brazil. The aim was to evaluate the activity of IMR and comparators against urinary, intra-abdominal, and respiratory tract isolates collected from patients over 18 years old during 2020–2021. The comparators used in the present study were a wide range of β**-**lactam antibacterial agents and β**-**lactam–β-lactamase inhibitor combinations such as amikacin (AMK), aztreonam (ATM), cefepime (FEP), ceftazidime (CAZ), ceftazidime/avibactam (CZA), ceftolozane/tazobactam (C/T), ceftriaxone (CRO), colistin (COL), ertapenem (ETP), IPM, levofloxacin (LVX), meropenem (MEM), and piperacillin/tazobactam (TZP).

## MATERIALS AND METHODS

### Clinical bacterial isolates

During the 2020–2021 period, as part of the SMART surveillance program, non-duplicate isolates were collected from 10 study sites located in six Brazilian cities. These cities included São Paulo, which hosted four sites, Rio de Janeiro with two sites, and Belo Horizonte, Curitiba, Recife, and Salvador, each having one site. *E. coli* and *K. pneumoniae* were isolated from intra-abdominal infections (IAI), lower respiratory tract infections, and urinary tract infection (UTI) specimens. All isolates were tested for organism identity using matrix-assisted laser desorption ionization-time-of-flight mass spectrometry (Bruker Daltonics, Billerica, MA). Only bacterial isolates determined to be significant as the reported probable cause of infection by local clinical and/or microbiological criteria were included in this investigation.

### Susceptibility testing

Antimicrobial susceptibility testing for AMK, ATM, FEP, CPZ, CZA, C/T, CRO, CIP, COL, ETP, IPM, IMR, MEM, and TZP was determined by the CLSI reference broth microdilution method (25, 26) using broth microdilution panels prepared at the International Health Management Associates and interpreted following Brazilian Committee on Antimicrobial Susceptibility (BrCAST)/EUCAST (27, 28) guidelines. Quality control (QC) of broth microdilution panels followed CLSI guidelines using the following strains: *E. coli* ATCC 25922, *K. pneumoniae* ATCC 700603, and *K. pneumoniae* BAA 2814. The corresponding QC values were within the acceptable ranges, as specified by the CLSI. *E. coli* and *K. pneumoniae* isolates with minimum inhibitory concentrations (MICs) ≥ 2 µg/mL for CAZ, CRO, or ATM were screened as “extended-spectrum β-lactamase (ESBL) phenotype.” *Enterobacterales* with an MIC ≥ 4 µg/mL for IPM and/or MEM were defined as carbapenem-resistant.

*Enterobacterales* isolates were considered MDR when resistant to ≥3 of the following compounds: AMK, IPM, FEP, CAZ, LVX, COL, ATM, and TZP.

### Molecular characterization of β-lactamase-encoding genes

Isolates meeting the following phenotypic criteria were screened for β-lactamase genes: NME isolates (excluding *Serratia* spp.) testing with IPM or IMR MIC values of ≥2 mg/L; *Enterobacterales* isolates testing with C/T MIC values of ≥4 mg/L. Previously published multiplex polymerase chain reaction assays were used to screen for the following β-lactamase enzyme encoding genes in *Enterobacterales*: ESBLs (CTX-M, GES, PER, SHV, TEM, VEB); acquired AmpC β-lactamases (ACC, ACT, CMY, DHA, FOX, MIR, MOX); serine carbapenemases (GES, KPC, OXA-48-like); and metallo-β-lactamases (including GIM, IMP, NDM, SPM, and VIM) (29). All detected acquired β-lactamase genes were re-amplified using gene-flanking primers and sequenced in full (Sanger), with the exception that limited sequencing was performed on *bla*_TEM_ and *bla*_SHV_ to identify genes encoding TEM-type and SHV-type enzymes containing amino acid substitutions common to ESBLs (SHV A146 V, G238S, G238A, E240 K, TEM E104 K, R164S, R164C, R164H, and G238S). Limited sequencing was also performed on *bla*_CTX-M_ to identify the presence of the D240G substitution in the deduced amino acid sequence associated with increased CAZ hydrolysis.

## RESULTS

**Fig 1:**
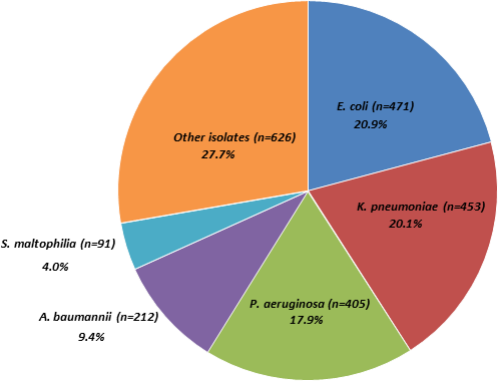
Distribution of isolates according to bacterial species collected from participating Brazilian study centers in the SMART program (Brazil, 2020–2021).

A total of 2,258 clinical isolates were collected from 10 study sites in Brazil. Among all isolates, the highest number were *Enterobacterales* such as *E. coli* (*n* = 471; 20.9%) and *K. pneumoniae* (*n* = 453; 20.1%), followed by *P. aeruginosa* (*n* = 405; 17.9%) and *A. baumannii* (*n* = 212; 9.4%) (Fig. 1).

The results from susceptibility testing are shown in Table 1. A total of 471 *E. coli* isolates were tested. The overall susceptibility rates for various antimicrobial agents were as follows: AMK 97.7% (*n* = 460), ATM 82.6% (*n* = 389), FEP 84.7% (*n* = 399), CAZ 86.0% (*n* = 405), CZA 99.4% (*n* = 468), C/T 98.5% (*n* = 464), CRO 75.8% (*n* = 357), COL 99.8% (*n* = 470), ETP 97.2% (*n* = 458), IPM 98.9% (*n* = 466), IMR 99.4% (*n* = 468), LEV 67.1% (*n* = 316), MEM 99.4% (*n* = 468), and TZP 92.8% (*n* = 437). Among the 70 MDR *E. coli* isolates, the susceptibility rates were significantly lower for most agents: AMK 94.3% (*n* = 66), ATM 2.9% (*n* = 2), FEP 8.6% (*n* = 6), CAZ 10.0% (*n* = 7), CZA 95.7% (*n* = 67), C/T 90.0% (*n* = 63), CRO 0% (*n* = 0), COL 98.6% (*n* = 69), ETP 90.0% (*n* = 63), IPM 92.9% (*n* = 65), IMR 95.7% (*n* = 67), LEV 25.7% (*n* = 18), MEM 95.7% (*n* = 67), and TZP 72.9% (*n* = 51).

**TABLE 1:** Antimicrobial susceptibility of *E. coli* and *K. pneumoniae* isolates, and subsets of MDR and pan-β-lactam-non-susceptible isolates^*a*^

Antimicrobial agent, % susceptible (BrCAST/EUCAST)															
		AMK	ATM	FEP	CAZ	CZA	C/T	CRO	COL	ETP	IPM	IMR	LEV	MEM	TZP
*E. coli*	N														
All	471	97.7	82.6	84.7	86.0	99.4	98.5	75.8	99.8	97.2	98.9	99.4	67.1	99.4	92.8
MDR	70	94.3	2.9	8.6	10.0	95.7	90.0	0	98.6	90.0	92.9	95.7	25.7	95.7	72.9
*K. pneumoniae*	N														
All	453	78.2	31.4	32.7	32.7	91.4	43.0	28.7	82.3	46.6	52.3	90.7	35.3	59.2	32.4
MDR	307	68.1	1.6	2.6	1.6	87.3	16.3	0.3	73.9	22.8	29.6	86.3	8.1	39.7	6.2
Pan-β-lactam—NS^*b*^	210	77.6	1.0	2.9	1.4	83.8	0.5	0	68.1	0	0.5	81.4	3.8	1.9	0

^*a*^ AMK, amikacin; ATM, aztreonam; FEP, cefepime; CAZ, ceftazidime; CZA, ceftazidime/avibactam; C/T, ceftolozane/tazobactam; CRO, ceftriaxone; COL, colistin; ETP, ertapenem; IPM, imipenem; IMR, imipenem/relebactam; LEV, levofloxacin; MEM, meropenem; TZP, piperacillin/tazobactam. MDR, multidrug resistant (resistant to ≥3 of the following compounds: AMK, IPM, FEP, CAZ, LVX, COL, ATM, and TZP).

^*b*^ Non-susceptible to ATM, FEP, CAZ, C/T, CRO, ETP, IPM, LEV, MEM, and TZP.

Among all the 453 *K*. *pneumoniae* tested, the overall susceptibility rates were AMK 78.2% (*n* = 354), ATM 31.4% (*n* = 142), FEP 32.7% (*n* = 148), CAZ 32.7% (*n* = 148), CZA 91.4% (*n* = 414), C/T 43.0% (*n* = 195), CRO 28.7% (*n* = 130), COL 82.3% (*n* = 373), ETP 46.6% (*n* = 211), IPM 52.3% (*n* = 237), IMR 90.7% (*n* = 411), LEV 35.3% (*n* = 160), MEM 59.2% (*n* = 268), and TZP 32.4% (*n* = 147). Among the 307 MDR *K. pneumoniae* isolates, the susceptibility rates were AMK 68.1% (*n* = 209), ATM 1.6% (*n* = 5), FEP 2.6% (*n* = 8), CAZ 1.6% (*n* = 5), CZA 87.3% (*n* = 268), C/T 16.3% (*n* = 50), CRO 0.3% (*n* = 1), COL 73.9% (*n* = 227), ETP 22.8% (*n* = 70), IPM 29.6% (*n* = 91), IMR 86.3% (*n* = 265), LEV 8.1% (*n* = 25), MEM 39.7% (*n* = 122), and TZP 6.2% (*n* = 19). Whereas, over 77% of the 210 β-lactam-non-susceptible *K. pneumoniae* isolates were susceptible to CZA (*n* = 176; 83.8%) and IMR (*n* = 171; 81.4%), in addition, they were non-susceptible to ATM, FEP, CAZ, C/T, CRO, ETP, IPM, LEV, MEM, and TZP.

Table 2 presents the frequency of β-lactamase class A and B among carbapenem-resistant *K. pneumoniae* isolates and new β-lactam/β-lactamase inhibitors (BL/BLI). Among the CZA-resistant *K. pneumoniae* isolates (*n* = 39), the genotype distribution observed for all carbapenemases was 66.7% (*n* = 26) *bla*_NDM-1_; 38.5% (*n* = 15) *bla*_KPC-2_; 15.4% (*n* = 6) *bla*_NDM-7_; *bla*_KPC-31_, and *bla*_NDM-5_. Most of them were susceptible to COL (*n* = 36; 92.3%) compared with 20.5% (*n* = 8) and 5.1% (*n* = 2) of isolates susceptible to MEM and IMR, respectively (Table 3). Also, most of the COL-resistant *K. pneumoniae* isolates presented susceptibility to CZA (*n* = 77; 96.3%) and IMR (*n* = 75; 93.8%) (Table 3).

**TABLE 2:** Frequency of β-lactamase class A and B among *K. pneumoniae* resistant to new BL/BLI^*a*^

Phenotype	No. of isolates (%)	Genotype distribution						
		NDM-1	NDM-5	NDM-7	KPC-31	KPC-2	KPC-3	VIM-1
CZA-R	39	26 (66.7%)	1 (2.6%)	6 (15.4%)	1 (2.6%)	15 (38.5%)	0 (0%)	0 (0%)
IMR-R	42	26 (61.9%)	1 (2.4%)	6 (14.3%)	0 (0%)	17 (40.5%)	0 (0%)	0 (0%)

^*a*^ CZA, ceftazidime/avibactam; IMR, imipenem/relebactam.

**TABLE 3:** Cross-susceptibility to CZA, COL, IR, and MEM among *K. pneumoniae* isolates with different phenotypes^*a*^

Phenotype	No. of isolates (%)	Susceptible + susceptible increased exposure			
		CZA	COL	IMR	MEM
CZA-R	39 (8.6%)	0	36 (92.3%)	2 (5.1%)	8 (20.5%)
COL-R	80 (17.7%)	77 (96.3%)	0	75 (93.8%)	17 (21.3%)
IMR-R	42 (9.2%)	5 (11.9%)	37 (88.1%)	0	6 (14.3%)
MEM-R	185 (40.8%)	154 (83.2%)	122 (66.0%)	149 (80.5%)	0

^*a*^ CZA, ceftazidime/avibactam; COL, colistin; IMR, imipenem/relebactam; MEM, meropenem.

The IMR-resistant *K. pneumoniae* isolates (*n* = 42) presented the following encoded gene distribution (Table 2): *bla*_NDM-1_ 61.9% (*n* = 26); *bla*_KPC-2_ 40.5% (*n* = 17); *bla*_NDM-7_ 14.3% (*n* = 6); and *bla*_NDM-5_ 2.4% (*n* = 1). The majority of the isolates were susceptible to COL (*n* = 37; 88.1%), and only five (11.9%) were susceptible to CZA.

**Fig 2:**
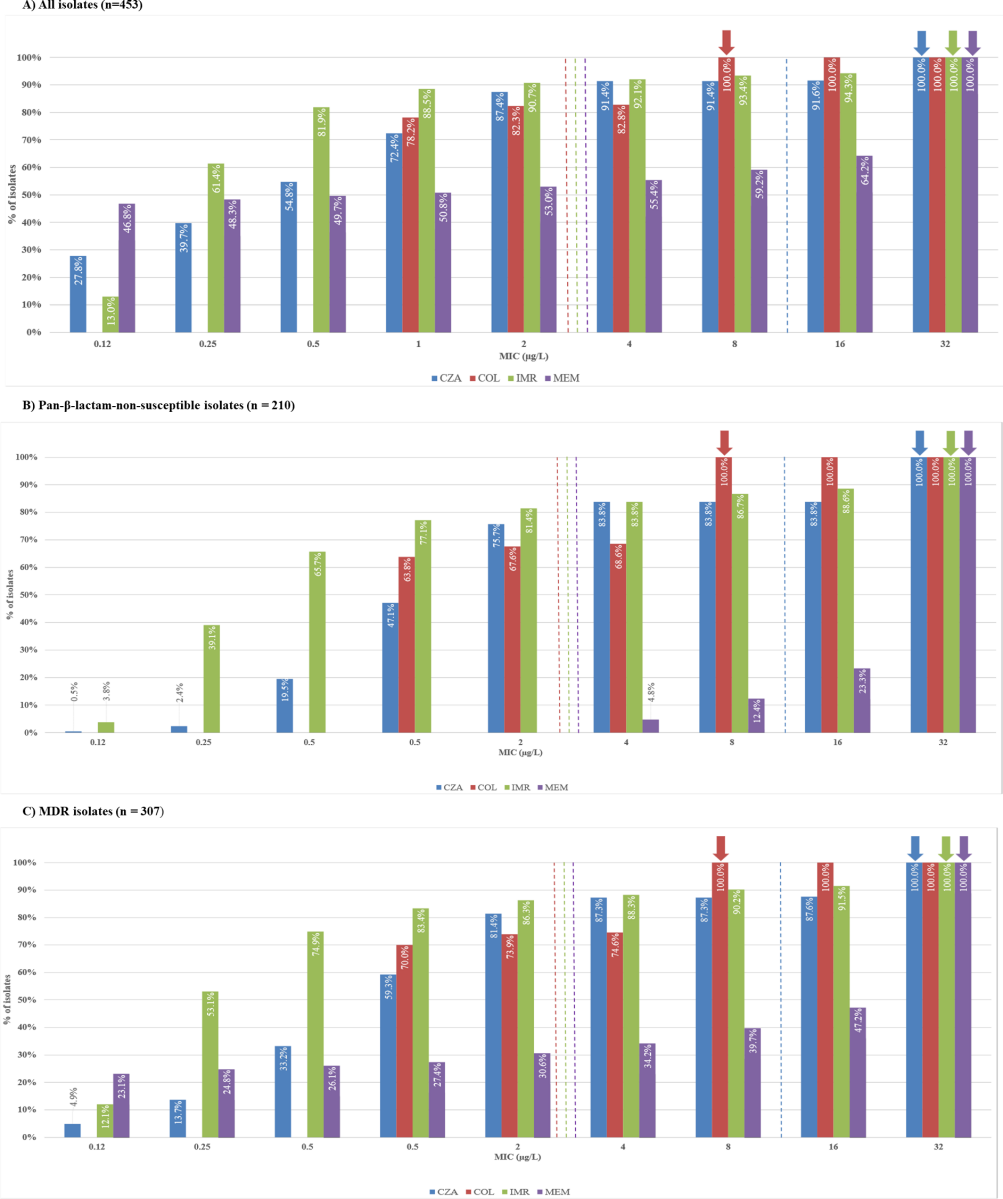
Distribution of CZA, COL, IMR, and MEM MIC values among *K. pneumoniae* isolates and non-susceptible subsets (collected from participating Brazilian study centers in the SMART program, 2020–2021). COL, colistin; IMR, imipenem/relebactam, MEM, meropenem; CZA, ceftazidime/avibactam; MDR, multidrug resistant (resistant to ≥3 of the following compounds: AMK, IPM, FEP, CAZ, LVX, COL, ATM, and TZP); MIC, minimum inhibitory concentration. Blue, red, green, and purple dotted lines represent breakpoint values for CZA, COL, IMR, and MEM, respectively; blue, red, green, and purple arrows represent MIC values for CZA, COL, IMR, and MEM, respectively. (**A**) All isolates (*n* = 453). (**B**) Pan-β-lactam-non-susceptible isolates (*n* = 210). (**C**) MDR isolates (*n* = 307).

Figure 2A through C provides MIC distributions for CZA, COL, IMR, and MEM for all the isolates and the isolates with specific antimicrobial-resistant phenotypes. The CZA, IMR, and MEM modal MICs were 32 mg/mL, each, whereas the COL modal MIC was 8 mg/mL for all isolates of *K. pneumoniae*. For pan-β-lactam-non-susceptible and MDR isolates, the modal MIC for CZA of 32 mg/mL was fourfold greater than its susceptible MIC breakpoint, and the modal MICs for IMR and MEM (32 mg/mL, each) were 16-fold greater than their susceptible MIC breakpoints. For pan-β-lactam-non-susceptible and MDR isolates, the modal MIC for COL of 8 mg/mL was fourfold greater than its susceptible MIC breakpoint.

## DISCUSSION

The widespread dispersion of MDR Gram-negative bacteria such as *Enterobacterales (E. coli* and *K. pneumoniae*) is an emerging challenge and poses a threat to the effective treatment of various infections (20). Management of infections caused by MDR *K. pneumoniae* is problematic due to the bacterium’s intrinsic and acquired resistance to a broad spectrum of drugs, such as β-lactams. β-lactamase-producing *K. pneumoniae* can destroy a varied range of β-lactams such as penicillins, carbapenems, and cephalosporins (30). According to the Infectious Diseases Society of America and other international consensus guidelines, the use of polymyxins and aminoglycosides is not recommended as the first-line treatment due to the associated nephrotoxicity (31, 32). The new β-lactam–β-lactamase inhibitor combinations such as CZA, meropenem/vaborbactam, and IMR are preferred treatment options for infections outside of the urinary tract caused by CRE (12, 31). Despite promising initial results and a preferable safety profile, resistance to CZA has emerged rapidly in *Enterobacterales* due to specific mutations within class A carbapenemases (1, 2, 9, 11).

Epidemiological surveillance studies monitor trends in antimicrobial susceptibility, provide clinical insights to guide treatment approaches progressively over time, and serve as an important tool that influences clinical decision-making and the choice of antibiotics for empirical treatment (24, 33). In the present study, conducted in Brazil from 2020 to 2021 as part of the SMART program, the microorganisms isolated from intra-abdominal, lower respiratory tract, and urinary tract samples were analyzed. The largest number of bacterial isolates were *Enterobacterales* such as *E. coli* and *K. pneumoniae,* consistent with previously published SMART studies (20, 22, 24).

We determined that >92.0% of MDR *E. coli* isolates were susceptible to IPM, indicating that these isolates most likely did not carry acquired carbapenemases. In this study, *K. pneumoniae* showed a higher resistance rate versus *E. coli*. This is corroborated from previous studies across multiple geographic regions and countries including Brazil, where *E. coli* has been found to be more susceptible than *K*. *pneumoniae* to IMP, CZA, and other antimicrobials (34, 35). The overall susceptibility of *K. pneumoniae* isolates to IMR was more than 90%. Previous studies describing the susceptibility of *Enterobacterales* among patients with IAIs and UTIs have reported similar susceptibility to IMR, as observed in the current study (5, 36, 37). We further determined that only 29.6% of MDR *K. pneumoniae* isolates were susceptible to IPM, whereas >86.0% of MDR *K. pneumoniae* isolates collected were susceptible to IMR, confirming a considerable enhancement in the susceptibility of IMR over IPM for these isolates. It also implies that MDR *K. pneumoniae* isolates probably carried acquired β-lactamase enzymes explaining their resistance to IPM. The European Centre for Disease Prevention and Control (EDCC) has reported that the rate of carbapenem resistance for *K. pneumoniae* and *E. coli* isolates reached 72.0% and 1.5% in 2022 in Greece, respectively (38). Another EDCC report on analysis of 874 *E. coli* isolates from the national collections of 13 countries in Europe, from 2012 to 2022, showed an increase of *E. coli* isolates carrying *bla*_NDM-5_, previously detected in carbapenem- and/or colistin-resistant *Enterobacterales* (CCRE-survey) (39). A study involving testing of 27,834 *Enterobacterales* isolates from 74 US hospitals determined that CRE comprised 0.9% (261) of the isolates, while an increase in prevalence was noted in 2021 (1.1%) compared to 2019 (0.8%) and 2020 (0.9% *P* = 0.06). The increase in CREs was driven by an increase in MBL-producing isolates in 2020 (40). Han R et al. in a comprehensive study on carbapenemase phenotype of isolates from patients, including adults and children in China, determined that *bla*_KPC-2_ (51.6%) and *bla*_NDM_ (35.7%) were the most common carbapenemase genes among CRE strains, with additional carbapenemase genes increasing recently (41).

Our study also showed that ≥90.0% of MDR *E. coli* isolates were susceptible to C/T, COL, AMK, IPM, IMR, CZA, MEM, and ETP. Consistent with other studies, COL, IPM, MEM, and AMK were the most active antimicrobials tested against *E. coli* in both IAIs (>97%) and UTIs (>99%) (42–45), and antimicrobial activity was almost similar during 2020–2021 when compared to the 2016–2017 SMART Spanish study (22). However, MDR *K. pneumoniae* isolates showed a decrease in the activity of carbapenems and AMK in comparison to 2016–2017 (22). Furthermore, we observed that the activity of IMR (86.3%) was similar to that of CZA (87.3%), but better than that of COL (73.9%) against MDR *K. pneumoniae* isolates. Similarly, the activity of IMR (81.4%) was almost the same as that of CZA (83.8%), but better than that of COL (68.1%) against pan β-lactam-non-susceptible isolates. Hence, the activity of IMR against pan-β-lactam-non-susceptible and against MDR *K. pneumoniae* isolates was 45%–80% percentage points higher than the activity of the commonly prescribed antimicrobials tested. Importantly, neither relebactam nor IPM is a substrate of resistance-nodulation-cell division efflux pumps that when upregulated, impact the effectiveness of a few other antipseudomonal β-lactams and β-lactam–β-lactamase combinations (46).

We also analyzed cross-susceptibility to CZA, COL, and IMR among *K. pneumoniae* isolates. Among CZA-resistant isolates, a greater percentage of isolates were COL susceptible (92.3%) than IMR susceptible (35.1%). Among colistin-non-susceptible isolates, a subtle but notable difference was observed between CZA (96.3% susceptible) and IMR (93.8% susceptible); among IMR-resistant isolates, COL was more active (88.1%) than CZA (11.9%). We also observed that modal MIC values for IMR against pan-β-lactam-non-susceptible and MDR isolates were 16-fold higher than their susceptible MIC breakpoints, while the modal MICs for CZA and COL were fourfold and four to eightfold higher than their susceptible MIC breakpoints, respectively. Although the corresponding annual percent susceptible MIC values for each agent did not change significantly over previous years, MICs do not completely predict the potency of antimicrobials. MIC is compared to the breakpoint; if the MIC is less than the breakpoint, the isolate is susceptible, while if the MIC is higher than the breakpoint, it is resistant (47). The SMART study from China reported that the addition of relebactam reduced the modal MICs of IPM by 64-fold for IPM-non-susceptible *Enterobacterales* (37). In addition, previous studies have reported that IMR had the greatest impact on *K. pneumoniae* expressing KPC and ESBLs (48, 49).

### Conclusion

This study reported the activity of IMR and comparators based on *in vitro* testing in the SMART program (2020–2021) for clinical isolates of *E. coli* and *K. pneumoniae*. IMR was one of the most effective β-lactam–β-lactamase inhibitor combinations tested and demonstrated comparable activity to CZA and higher activity than COL against both the *Enterobacterales* species tested. In summary, considering the high susceptibility of IMR against *Enterobacterales* infections, it provides an important new treatment option for patients in Brazil, especially against isolates that are not susceptible to conventional carbapenems and MDR isolates. Moreover, in the future, the shift in susceptibility rates of *Enterobacterales* to carbapenems should be monitored actively for effective treatment of such infections.

## Data Availability

All data analyses were performed in Excel (Microsoft, Redmond, WA) attached as a supplemental file, SMART Data.

## SUPPLEMENTAL MATERIAL

SMART Data spectrum.02543-24-s0001.xlsx SMART Data.

ASM does not own the copyrights to Supplemental Material that may be linked to, or accessed through, an article. The authors have granted ASM a non-exclusive, world-wide license to publish the Supplemental Material files. Please contact the corresponding author directly for reuse.
